# Using the Reverse Shock Index at the Injury Scene and in the Emergency Department to Identify High-Risk Patients: A Cross-Sectional Retrospective Study

**DOI:** 10.3390/ijerph13040357

**Published:** 2016-03-24

**Authors:** Wei-Hung Lai, Cheng-Shyuan Rau, Shiun-Yuan Hsu, Shao-Chun Wu, Pao-Jen Kuo, Hsiao-Yun Hsieh, Yi-Chun Chen, Ching-Hua Hsieh

**Affiliations:** 1 Department of Trauma Surgery, Kaohsiung Chang Gung Memorial Hospital and Chang Gung University College of Medicine, Kaohsiung 833, Taiwan; abdiel@cgmh.org.tw (W.-H.L.); ah.lucy@hotmail.com (S.-Y.H.); sylvia19870714@hotmail.com (H.-Y.H.); libe320@yahoo.com.tw (Y.-C.H.); 2 Department of Neurosurgery, Kaohsiung Chang Gung Memorial Hospital and Chang Gung University College of Medicine, Kaohsiung 833, Taiwan; ersh2127@cloud.cgmh.org.tw; 3 Department of Anesthesiology, Kaohsiung Chang Gung Memorial Hospital and Chang Gung University College of Medicine, Kaohsiung 833, Taiwan; shaochunwu@gmail.com; 4 Department of Plastic and Reconstructive Surgery, Kaohsiung Chang Gung Memorial Hospital and Chang Gung University College of Medicine, Kaohsiung 833, Taiwan; bow110470@gmail.com

**Keywords:** reverse shock index (RSI), shock index (SI), emergency medical services (EMS), injury severity score (ISS), length of stay (LOS), mortality

## Abstract

*Background*: The ratio of systolic blood pressure (SBP) to heart rate (HR), called the reverse shock index (RSI), is used to evaluate the hemodynamic stability of trauma patients. A SBP lower than the HR (RSI < 1) indicates the probability of hemodynamic shock. The objective of this study was to evaluate whether the RSI as evaluated by emergency medical services (EMS) personnel at the injury scene (EMS RSI) and the physician in the emergency department (ED RSI) could be used as an additional variable to identify patients who are at high risk of more severe injury. *Methods*: Data obtained from all 16,548 patients added to the trauma registry system at a Level I trauma center between January 2009 and December 2013 were retrospectively reviewed. Only patients transferred by EMS were included in this study. A total of 3715 trauma patients were enrolled and subsequently divided into four groups: group I patients had an EMS RSI ≥1 and an ED RSI ≥1 (*n* = 3485); group II an EMS RSI ≥ 1 and an ED RSI < 1 (*n* = 85); group III an EMS RSI < 1 and an ED RSI ≥ 1 (*n* = 98); and group IV an EMS RSI < 1 and a ED RSI < 1 (*n* = 47). A Pearson’s χ^2^ test, Fisher’s exact test, or independent Student’s t-test was conducted to compare trauma patients in groups II, III, and IV with those in group I. *Results*: Group II and IV patients had a higher injury severity score, a higher incidence of commonly associated injuries, and underwent more procedures (including intubation, chest tube insertion, and blood transfusion in the ED) than patients in group I. Group II and IV patients were also more likely to receive a severe injury to the thoracoabdominal area. These patients also had worse outcomes regarding the length of stay in hospital and intensive care unit (ICU), the proportion of patients admitted to ICU, and in-hospital mortality. Group II patients had a higher adjusted odds ratio for mortality (5.8-times greater) than group I patients. *Conclusions*: Using an RSI < 1 as a threshold to evaluate the hemodynamic condition of the patients at the injury scene and upon arrival to the ED provides valid information regarding deteriorating outcomes for certain subgroups of patients in the ED setting. Particular attention and additional resources should be provided to patients with an EMS RSI ≥ 1 that deteriorates to an RSI < 1 upon arrival to the ED since a higher odds of mortality was found in these patients.

## 1. Background

Acute trauma patients presenting with shock in the prehospital or emergency department (ED) setting need focused care. Measurement of vital signs, including systolic blood pressure (SBP), is often included in the initial triage for acute trauma patients and is recommended by many clinical guidelines as a basic part of the initial assessment of circulatory blood volume by emergency medical services (EMS) personnel. In addition to the trauma cause, a description of the hemodynamic status, including its severity, provided by responsible EMS personnel could be an important reference for the emergency physician, who could use this information to provide adequate patient care [1,2].

Hypovolemic shock is the most common type of shock in patients who experience traumatic injury. In a meta-analysis of six observational studies, the prevalence of hypotensive shock in the prehospital setting was between 9.5 and 19 per 1000 EMS contacts with an in-hospital shock mortality between 33% and 52% [3]. In addition, the prevalence of hypotension in the ED was 4–13/1000 ED contacts with a mortality of 12% [3]. To identify hypovolemic shock, isolated vital signs, such as SBP and heart rate (HR), have been shown to be unreliable [4,5]. For example, individuals who are habitually hypertensive can have a normal blood pressure during shock, and hypotensive individuals can have normal tissue perfusion [6,7]. Some observational studies conducted in the ED and prehospital setting with hypotensive patients who experienced a trauma have advocated for a higher SBP threshold to correspond with the actual mortality rate [8,9,10]. The shock index (SI), the ratio of HR to SBP, has been assessed as a marker of significant injury in trauma patients with hypovolemic shock [11,12,13], and been found to be more useful in predicting early shock than either the HR or the SBP alone and to correlate with other indices of end-organ perfusion (such as central venous oxygen saturation and arterial lactic acid concentration) [14]. The SI has been demonstrated to be a capable measure for hemodynamic instability [15,16,17] and a useful guide for diagnosing early acute hypovolemia in the presence of normal HR and blood pressure [18]. It has also been shown to be a clinical indicator of hypovolemic shock upon arrival to the ED with respect to transfusion requirements and hemostatic resuscitation [19].

An elevated SI > 0.7 is correlated with reduced left ventricular end-diastolic pressure [20] and reductions in circulatory blood volume [13] even when the pulse rate and SBP remain in the normal range. A pre-intubation SI greater than or equal to 0.8 is an independent risk factor for cardiovascular deterioration after emergency intubation in the ED [21]. An SI that exceeds 0.9 between the field and the ED may predict higher mortality [13]. The sensitivity and specificity of an SI greater than 0.9 as a predictor of massive transfusion (defined as 24-h red blood cell transfusion ≥9 units) are 63% and 83%, respectively [22]. An SI greater than or equal to 1 is associated with an adjusted odds ratio (AOR) of 10.5 (95% confidence interval (CI): 9.3–11.7) for 30-day mortality [23]. In addition, patients with an SI ≥ 1.0, despite prehospital crystalloid resuscitation, had a significantly higher transfusion requirement and a higher mortality rate than other major trauma patients [15]. Furthermore, an SI greater than 1.4 has been proposed as a more practical cutoff for predicting massive transfusion in trauma patients [22].

Although the SI is a very practical and useful predictor of outcomes for trauma patients, the literature has shown that the relevant SI cut-off point varies depending on the cause of trauma [12,13,20,23,24] and patient’s illness [23]. Moreover, the calculation of the SI as the ratio of HR to SBP appears contradictory to the basic concept of shock, which is generally thought of as an unstable hemodynamic status in which the SBP is lower than the HR, and not as the SI, which indicates that the HR is higher than the SBP. Therefore, the reverse shock index (RSI), the ratio of SBP to HR, is often preferred to evaluate the hemodynamic stability of trauma patients. An RSI < 1 indicates that the SBP is lower than the HR, and implies that the patient is probably in shock. Moreover, the RSI could be assessed without any additional calculation or equipment by first responders upon arrival at the site of injury or to the ED.

Identification of patients with shock is crucial as prompt treatment improves the prognosis and the optimal treatment differs depending on the cause [7]. The objective of this study was to evaluate whether an RSI evaluated by EMS personnel at the scene of injury (EMS RSI) and the physician at the ED (ED RSI) could be used to identify patients who are at high risk for more severe complications. To accomplish this, we used data from a trauma registry system collected over a five-year period at a Level I trauma center.

## 2. Methods

### 2.1. Ethics Statement

The hospital’s institutional review board (IRB) approved this study (approval number 104-0578B). Informed consent was waived according to the IRB regulations.

### 2.2. Study Design

**Figure 1:**
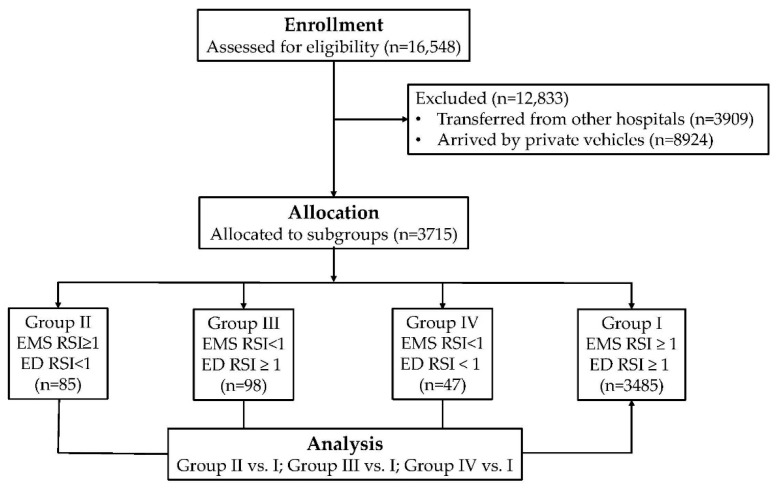
Flow diagram of studied groups of patients.

This retrospective study was conducted at the Kaohsiung Chang Gung Memorial Hospital, a 2400-bed facility and Level I regional trauma center that provides care to trauma patients primarily from southern Taiwan. This study reviewed all 16,548 hospitalized and registered patients added to the Trauma Registry System between 1 January 2009 and 31 December 2013 (Figure 1). During this time, only patients who were transferred by EMS were included in this study. Those who were transferred from other hospitals or arrived by private vehicles were not included in the study population. Patients who had incomplete data were also excluded. The RSI was calculated as the ratio of SBP to HR (RSI = SBP/HR). In total, 3715 trauma patients were enrolled in this study. These patients were then divided into 4 groups. Group I included those with an EMS RSI ≥ 1 and an ED RSI ≥ 1 (*n* = 3485); group II included those with an EMS RSI ≥ 1 and an ED RSI < 1 (*n* = 85); group III those with an EMS RSI < 1 and an ED RSI ≥ 1 (*n* = 98); and group IV those with an EMS RSI < 1 and an ED RSI < 1 (*n* = 47). Group I (patients with a stable hemodynamic status at the injury scene and in the ED) was used as a reference for comparison with group II (patients with a stable hemodynamic status at the injury scene but who got worse upon arrival to the ED), group III (patients with hemodynamic instability at the injury scene but who improved upon arrival to the ED), and group IV (patients with hemodynamic instability at the injury scene and in the ED). The vital signs of the patients generally would be measured within 5 min upon arrival to the ED.

Detailed patient information regarding age, sex, vital signs (assessed by the EMS personnel at the injury scene and by the physician upon arrival to the ED), the Glasgow coma scale (GCS) score (assessed upon arrival to the ED), procedures performed by EMS personnel at the injury scene (intubation, airway placement, neck collar placement, backboard or spinal immobilization, oxygenation, and cardiopulmonary resuscitation) and by the physician in the ED (intubation, chest tube insertion, and blood transfusion), the abbreviated injury scale (AIS) score for each body region, the injury severity score (ISS), the new injury severity score (NISS), the trauma and injury severity score (TRISS), length of stay (LOS) in the hospital, LOS in the intensive care unit (ICU), in-hospital mortality, and complications associated with injuries. In our study, the primary outcomes were in-hospital mortality and injury severity as measured by different scoring systems (GCS, AIS, ISS, NISS, and TRISS). The secondary outcomes were LOS in the hospital and in the ICU.

Data were compared using SPSS version 20 statistical software (IBM Corporation, Armonk, NY, USA). We used a Pearson’s χ^2^ test, Fisher’s exact test, or independent Student’s *t*-test, as applicable. The Mann Whitney U test was used to compare the LOS in hospital and ICU. All results are presented as a mean ± standard error. A *p*-value of <0.05 was considered as statistically significant. Odds ratios (ORs) were calculated with 95% confidence intervals (CIs). AORs for mortality adjusted by the ISS with 95% CIs were also calculated.

## 3. Results

### 3.1. Patient Injury Characteristics

The mean age of patients in groups I, II, III, and IV were 41.9 ± 14.4, 36.6 ± 13.5, 39.2 ± 15.5, and 38.9 ± 12.5 years, respectively (Table 1). Compared to patients in group I, patients in group II were younger (*p* = 0.001), and statistically significant differences regarding sex were found between group II patients (men: *n* = 60 (70.6%); women: *n* = 25 (29.4%)) and group IV patients (men: *n* = 36 (76.6%); women: *n* = 11 (23.4%)) when compared to group I patients (men: *n* = 2026 (58.1%); women: *n* = 1459 (41.9%)). There were also significantly lower GCS scores in both group II (12.4 ± 3.8) and group IV (13.0 ± 3.3) than in group I (14.3 ± 2.1). In addition, the distribution of GCS scores (≤8, 9–12, or ≥13) were different in group II and IV patients than group I patients. The analysis of AIS scores revealed that group II patients sustained significantly higher rates of injuries to the head/neck, thorax, and abdomen, but lower rates of injuries to the extremities than group I patients; group III patients sustained significantly higher rates of injuries to the thorax; and group IV patients sustained significantly higher rates of injuries to the head/neck, thorax, and abdomen. A significantly higher ISS was found in group II (15.1 ± 11.1; *p* < 0.001), group III (10.5 ± 7.1; *p* = 0.026), and group IV (15.8 ± 11.3; *p* < 0.001) in comparison to group I (8.9 ± 6.9). When stratified by ISS (<16, 16–24, or ≥25), group II included more patients with an ISS ≥ 25 and less patients with an ISS < 16 than in group I while group IV included more patients with an ISS ≥ 25 and an ISS between 16 and 24 and less patients with an ISS of <16 than group I. No differences were found between group III and group I regardless of the stratification by ISS (<16, 16–24, or ≥25). Likewise, we also found a significantly higher NISS in group II (17.8 ± 14.4; *p* < 0.001), group III (12.3 ± 8.3; *p* = 0.036), and group IV (16.7 ± 12.6; *p* < 0.001) than in group I (10.5 ± 8.5). When compared with the TRISS of group I (0.991 ± 0.093), a significantly lower TRISS, indicating a lower survival rate, was found in group II (0.953 ± 0.213; *p* < 0.001) and group IV (0.957 ± 0.204; *p* < 0.001), but not in group III (0.980 ± 0.142; *p* = 0.233). The in-hospital mortality rates for group I, II, III, and IV were 0.9%, 12.9%, 1.0%, and 4.3%, respectively. After adjusting for ISS, the AOR for mortality for patients in group II (AOR = 5.8, 95% CI: 2.3–14.4, *p* < 0.001) and IV (AOR = 1.1, 95% CI: 1.1–1.1, *p* < 0.001) was significantly greater than that of patients in group I. No difference in the AOR for mortality was found between group III and I.

### 3.2. Management Characteristics

There were no significant differences regarding the transport times for group II, III, and IV compared to group I (Table 2). Regarding the procedures performed by the EMS personnel, more group II patients underwent airway placement, placement of a neck collar, backboard and spinal immobilization, an oxygen supplement, and cardiopulmonary resuscitation than group I patients; more group III patients underwent airway placement, backboard immobilization, and an oxygen supplement; and more group IV patients underwent placement of a neck collar, backboard immobilization, and an oxygen supplement. Regarding the procedures performed in the ED, more group II and group IV patients underwent intubation, chest tube insertion, and blood transfusion than group I patients. No significant differences regarding the procedures performed in the ED were noted between group III and I patients.

### 3.3. Associated Injuries

As shown in the Table 3, the OR for sustaining intracerebral hematoma (OR: 3.8, 95% CI: 1.6–8.9, *p* = 0.001), a cervical vertebral fracture (OR: 4.5, 95% CI: 1.4–15.2, *p* = 0.007), pneumothorax (OR: 3.0, 95% CI: 1.1–8.4, *p* = 0.031), hemopneumothorax (OR: 6.2, 95% CI: 2.4–16.1, *p* < 0.001), a lung contusion (OR: 4.0, 95% CI: 1.4–11.3, *p* = 0.006), hepatic injury (OR: 8.0, 95% CI: 4.2–15.4, *p* < 0.001), splenic injury (OR: 6.1, 95% CI: 2.1–17.8, *p* < 0.001), retroperitoneal injury (OR: 16.8, 95% CI: 3.2–87.7, *p* < 0.001), and renal injury (OR: 6.4, 95% CI: 1.4–29.0, *p* = 0.005) was statistically significantly higher for group II patients than group I patients. Similarly, the OR for sustaining a rib fracture (OR: 1.8, 95% CI: 1.1–3.1, *p* = 0.023), hemothorax (OR: 4.7, 95% CI: 2.0–11.42, *p* < 0.001), and hepatic injury (OR: 2.6, 95% CI: 1.0–6.7, *p* = 0.035) was statistically significantly higher for group III patients than group I patients. Group IV patients had a statistically significantly higher OR for sustaining a rib fracture (OR: 3.7, 95% CI: 2.0–7.0, *p* < 0.001), hemothorax (OR: 6.7, 95% CI: 2.3–19.3, *p* < 0.001), pneumothorax (OR: 4.1, 95% CI: 1.2–13.6, *p* = 0.012), hemopneumothorax (OR: 14.4, 95% CI: 5.8–36.2, *p* < 0.001), an intra-abdominal injury (OR: 7.7, 95% CI: 3.4–17.8, *p* < 0.001), hepatic injury (OR: 8.5, 95% CI: 3.7–19.7, *p* < 0.001), splenic injury (OR: 11.5, 95% CI: 3.9–34.2, *p* < 0.001), and pelvic fracture (OR: 4.9, 95% CI: 2.1–11.1, *p* < 0.001) than group I patients.

### 3.4. LOS in Hospital and ICU

A significantly longer hospital LOS was found among group II and IV patients compared with group I patients (15.2 and 16.9 *vs.* 9.5 days, respectively; *p* < 0.001) (Table 4). Likewise, a significantly larger proportion of group II and IV patients than group I patients were admitted to the ICU (45.9% and 51.1% *vs.* 16.5%, respectively; *p* < 0.001), but the ICU LOS was not significantly longer (13.1 and 13.0 *vs.* 7.6 days, respectively; *p* = 0.296 and *p* = 0.070, respectively).

## 4. Discussion

In this study, group II patients (*i.e.*, those who had a stable hemodynamic status as evaluated by EMS personnel at the injury scene but got worse upon arrival to the ED) and group IV patients (*i.e.*, those who had an unstable hemodynamic condition both at the injury scene and upon arrival to the ED) had worse outcomes than group I patients (*i.e.*, those who had a stable hemodynamic condition at the injury scene and in the ED). Group II and IV patients had a higher ISS, a higher incidence of commonly associated injuries, and underwent more procedures than group I patients. These patients also had worse outcomes regarding hospital LOS, the proportion of patients admitted to the ICU, and in-hospital mortality. On the other hand, group III patients (*i.e.*, those who had an improved hemodynamic condition upon arrival to the ED than that at the injury scene), had a good response after EMS involvement and demonstrated no significant differences regarding injury severity, mortality, the proportion of patients admitted to the ICU, and hospital and ICU LOS when compared to group I patients.

Therefore, specific attention should be provided to group II patients because they have a higher AOR for mortality than patients in other groups. For example, group II patients had an AOR for mortality 5.8-times greater than that of group I patients while group IV patients had an AOR for mortality 1.1-times greater than group I patients. Group II patients are younger and sustained injuries to more regions (excluding the face) than group I patients. Considering that the mean transport time was short (less than 25 min) in all groups, the quickly deteriorating hemodynamic condition upon arrival to the ED is an alarming sign for these relatively younger patients. In particular, these patients are more likely to have a severe injury to the thoracoabdominal areas. A higher OR was also found for sustaining pneumothorax (3.0-fold), hemopneumothorax (6.2-fold), a lung contusion (4.0-fold), hepatic injury (8.0-fold), splenic injury (6.1-fold), retroperitoneal injury (16.8-fold), and renal injury (6.4-fold) among group II patients when compared with group I patients. In addition, a higher OR for intubation (7.7-fold), chest tube insertion (9.5-fold), and blood transfusion (12.1-fold) was noted in group II patients as well.

Furthermore, group IV and group II patients had similar characteristics and a similar injury pattern. Group IV patients had a similarly high ISS as well as a higher OR for sustaining a rib fracture (3.7-fold), hemothorax (6.7-fold), pneumothorax (4.1-fold), hemopneumothorax (14.4-fold), an intra-abdominal injury (7.7-fold), hepatic injury (8.5-fold), splenic injury (11.5-fold), and pelvic fracture (4.9-fold) when compared to group I patients. In addition, a higher OR for intubation (5.9-fold), chest tube insertion (9.5-fold), and blood transfusion (17.3-fold) was also found. However, the AOR for mortality was only slightly significantly higher than that of group I patients (AOR: 1.1, 95% CI: 1.1–1.1, *p* < 0.001), even though hemodynamic instability was established at both the injury scene and in the ED. No strong evidence defining the optimal blood pressure level during active hemorrhagic shock has been documented in the literature [25,26] and the optimal blood pressure level during resuscitation of a hemorrhagic shock patient is still debated. One study proposed administering a limited amount of fluids before bleeding control to maintain arterial pressure and minimize dilution of coagulation factors as well as complications from over fluid resuscitation [27]. For patients with trauma but without brain injury, European guidelines recommend a target SBP of 80–90 mmHg until major bleeding in the initial phase has been stopped [28]. Moreover, a policy of permissive hypotension with judicious fluid administration to maintain a mean arterial pressure in the 60–80 mmHg range is advisable and appropriate [29,30,31]. Notably, the significantly higher incidence of hemothorax and pelvic fracture in group IV patients accompanied by massive blood loss had to be treated with blood or fluid transfusions as well as more advanced procedures (*i.e.*, chest tube insertion or pressure garment compression, respectively). The higher mortality rate found in group II patients compared to group IV patients indicates that patients who have a stable hemodynamic condition at the injury scene but experience a rapid deterioration of their hemodynamic status require specific attention. Young patients who present with tachycardia and mild hypotension are in danger of compensatory mechanism failure and may slip into profound shock unless vigorous therapy is initiated [14]. Reliance on SBP alone may delay recognition of the shock state [14].

Traumatic injury remains the leading cause of death among people less than 44 years old and 40% of trauma-related deaths are caused by uncontrolled hemorrhagic shock or its sequelae (such as multiple organ failure) [32]. Significant, untapped opportunities for early recognition and treatment of critical illnesses may exist within the EMS population. Assessment and treatment of trauma patients upon arrival to the ED is essential in the presence of life-threatening injuries. Prospectively identifying patients that would benefit from trauma care is essential to the success of trauma systems. Although little is known about the ability of EMS personnel to accurately approximate the volume of blood loss in an out-of-hospital setting, previous studies have reported that EMS providers are not able to adequately estimate spilled blood volumes [33]. Following the advanced trauma life support paradigm, “keep algorithms simple”, an RSI < 1 may serve as an alert for attending physicians in the ED. Given the circumstances in an ED (e.g., many patients waiting in crowded EDs for hours before a physician evaluation) [34], the timely recognition and rapid treatment of shock can be a difficult task. One of the major benefits of using the RSI for evaluation in the ED is that it can be used quickly when first responders arrive without requiring any additional equipment or cost. An RSI < 1 can alert trauma surgeons to the need for early intervention and timely preparation upon the arrival of the patient. In particular, attention should be placed on those with an EMS RSI ≥ 1 that deteriorates in the ED. Using the EMS RSI as an additional information may also help alert physicians to a thoracoabdominal injury with massive bleeding and help to identify patients with serious injuries who need upgraded higher level of intervention. This may help improve patient outcomes after a severe injury.

Our analysis has several limitations. First, the data were collected prospectively as part of the required trauma registry process, but our analyses were performed retrospectively and are thus subject to the limitations of all retrospective studies. Second, injured patients who did not survive until arrival at the hospital or who were discharged from the ED were not included in the sample, which could result in a selection bias. Third, age, hypertension, and β- or calcium channel blockers weaken the association between the SI and mortality rate [23], and the impact of pre-existing comorbidities on the course of hospitalization and mortality was not included in the analysis and, thus, remains unclear. In addition, some missing data and the lack of available data regarding patient management, including type, volume, and speed of fluid resuscitation during transportation by EMS and in the ED, may result in a bias in the outcome. Finally, some important data, such as cost, treatment delays, and complications, were not evaluated and may have limited the outcome evaluation results.

## 5. Conclusions

This retrospective analysis spanning a five-year period showed that using an RSI < 1 as a threshold to evaluate the hemodynamic condition of trauma patients at the injury scene and upon arrival to the ED provides valid information regarding the deteriorating outcomes of certain subgroups of patients in the ED setting. Particular attention and resources should be provided for patients with an EMS RSI ≥ 1 that deteriorates to an RSI < 1 upon arrival to the ED since a higher odds of mortality was found in these patients.

## Figures and Tables

**Table 1:** Demographic and injury characteristics of hospitalized trauma patients.

Variables	EMS RSI ≥ 1 ED RSI < 1 *n* = 85 (II)	EMS RSI < 1 ED RSI ≥ 1 *n* = 98 (III)	EMS RSI < 1 ED RSI < 1 *n* = 47 (IV)	EMS RSI ≥ 1 ED RSI ≥ 1 *n* = 3485 (I)	*OR (95%CI) p*		*OR (95%CI) p*		*OR (95%CI) p*	
					II *vs*. I		III *vs*. I		IV *vs*. I	
Age	36.6 ± 13.5	39.2 ± 15.5	38.9 ± 12.5	41.9 ± 14.4	-	0.001	-	0.071	-	0.158
Gender						0.021		0.690		0.011
Male	60 (70.6)	55 (56.1)	36 (76.6)	2026 (58.1)	1.7 (1.1–2.8)		0.9 (0.6–1.4)		2.4 (1.2–4.7)	
Female	25 (29.4)	43 (43.9)	11 (23.4)	1459 (41.9)	0.6 (0.4–0.9)		1.1 (0.7–1.6)		0.4 (0.2–0.8)	
GCS	12.4 ± 3.8	14.2 ± 2.1	13.0 ± 3.3	14.3 ± 2.1	-	<0.001	-	0.669	-	<0.001
≤8	16 (18.8)	5 (5.1)	7 (14.9)	148 (4.2)	5.2 (3.0–9.2)	<0.001	1.2 (0.5–3.0)	0.680	3.9 (1.7–9.0)	<0.001
9–12	13 (15.3)	5 (5.1)	5 (10.6)	156 (4.5)	3.9 (2.1–7.1)	<0.001	1.1 (0.5–2.9)	0.768	2.5 (1.0–6.5)	0.044
≥13	56 (65.9)	88 (89.8)	35 (74.5)	3181 (91.3)	0.2 (0.1–0.3)	<0.001	0.8 (0.4–1.6)	0.609	0.3 (0.1–0.5)	<0.001
AIS										
Head/Neck	39 (45.9)	32 (32.7)	22 (46.8)	1074 (30.8)	1.9 (1.2–2.9)	0.003	1.1 (0.7–1.7)	0.698	2.0 (1.1–3.5)	0.019
Face	19 (22.4)	21 (21.4)	11 (23.4)	734 ( (21.1)	1.1 (0.6–1.8)	0.773	1.0 (0.6–1.7)	0.930	1.1 (0.6–2.3)	0.696
Thorax	29 (34.1)	23 (23.5)	23 (48.9)	477 (13.7)	3.3 (2.1–5.2)	<0.001	1.9 (1.2–3.1)	0.006	6.0 (3.4–10.8)	<0.001
Abdomen	21 (24.7)	10 (10.2)	19 (40.4)	237 (6.8)	4.5 (2.7–7.5)	<0.001	1.6 (0.8–3.0)	0.190	9.3 (5.1–16.9)	<0.001
Extremity	54 (63.5)	72 (73.5)	33 (70.2)	2585 (74.2)	0.6 (0.4–1.0)	0.027	1.0 (0.6–1.5)	0.875	0.8 (0.4–1.5)	0.538
ISS	15.1 ± 11.1	10.5 ± 7.1	15.8 ± 11.3	8.9 ± 6.9	-	<0.001	-	0.026	-	<0.001
<16	48 (56.5)	76 (77.6)	23 (48.9)	2794 (80.2)	0.3 (0.2–0.5)	<0.001	0.9 (0.5–1.4)	0.522	0.2 (0.1–0.4)	<0.001
16–24	16 (18.9)	16 (6.1)	14 (29.8)	483 (13.9)	1.4 (0.8–2.5)	0.192	1.2 (0.7–2.1)	0.487	2.6 (1.4–5.0)	0.002
≥25	21 (24.7)	6 (6.1)	10 (21.3)	208 (6.0)	5.2 (3.1–8.6)	<0.001	1.0 (0.5–2.4)	0.949	4.3 (2.1–8.7)	<0.001
NISS	17.8 ± 14.4	12.3 ± 8.3	16.7 ± 12.6	10.5 ± 8.5	-	<0.001	-	0.036	-	<0.001
TRISS	0.953 ± 0.213	0.980 ± 0.142	0.957 ± 0.204	0.991 ± 0.093	-	<0.001	-	0.233	-	0.016
Mortality	11 (12.9)	1 (1.0)	2 (4.3)	32 (0.9)	16.0 (7.8–33.0)	<0.001	1.1 (0.2–8.2)	0.917	4.8 (1.1–20.6)	0.020
ISS	-	-	-	-	5.8 (2.3–14.4)	<0.001	1.0 (1.0–1.1)	0.053	1.1 (1.1–1.1)	<0.001

**Table 2:** Transport time and procedures performed by EMS and ED personnel.

Variables	EMS RSI ≥ 1 ED RSI < 1 *n* = 85 (II)	EMS RSI < 1 ED RSI ≥ 1 *n* = 98 (III)	EMS RSI < 1 ED RSI < 1 *n* = 47 (IV)	EMS RSI ≥ 1 ED RSI ≥ 1 *n* = 3485 (I)	*OR (95%CI) p*		*OR (95%CI) p*		*OR (95%CI) p*	
					II *vs*. I		III *vs*. I		IV *vs*. I	
Transport time										
Mean (mins)	23.0 ± 9.6	24.2 ± 8.9	22.1 ± 6.7	22.8 ± 9.4	-	0.864	-	0.138	-	0.603
Range (mins)	10–67	12–68	10–36	4–142	-	-	-	-	-	-
Procedures performed by EMS personnel										
Intubation	0 (0.0)	0 (0.0)	0 (0.0)	0 (0.0)	-	-	-	-	-	-
Airway	2 (2.4)	4 (4.1)	1 (2.1)	17 (0.5)	4.9 (1.1–21.6)	0.020	8.7 (2.9–26.3)	<0.001	4.4 (0.6–34.0)	0.117
Neck collar	29 (34.1)	28 (28.6)	22 (46.8)	849 (24.4)	1.6 (1.0–2.5)	0.039	1.2 (0.8–1.9)	0.339	2.7 (1.5–4.9)	<0.001
Backboard	36 (42.4)	36 (36.7)	23 (48.9)	930 (26.7)	2.0 (1.3–3.1)	0.001	1.6 (1.1–2.4)	0.027	2.6 (1.5–4.7)	0.001
Spinal immobilizer	3 (3.5)	1 (1.0)	0 (0.0)	12 (0.3)	10.6 (2.9–38.2)	<0.001	3.0 (0.4–23.2)	0.272	1.0 (1.0–1.0)	0.687
Oxygenation	15 (17.6)	12 (12.2)	11 (23.4)	203 (5.8)	3.5 (2.0–6.2)	<0.001	2.3 (1.2–4.2)	0.008	4.9 (2.5–9.9)	<0.001
Cardiopulmonary resuscitation	1 (1.2)	0 (0.0)	0 (0.0)	1 (0.0)	41.5 (2.6–668.7)	<0.001	-	0.867	-	0.908
Procedures at ED										
Intubation	18 (21.2)	5 (5.1)	8 (17.0)	117 (3.4)	7.7 (4.5–13.4)	<0.001	1.5 (0.6–3.9)	0.348	5.9 (2.7–12.9)	<0.001
Chest tube insertion	9 (10.6)	3 (3.1)	5 (10.6)	43 (1.2)	9.5 (4.5–20.1)	<0.001	2.5 (0.8–8.3)	0.113	9.5 (3.6–25.3)	<0.001
Blood transfusion	21 (24.7)	4 (4.1)	15 (31.9)	92 (2.6)	12.1 (7.1–20.7)	<0.001	1.6 (0.6–4.4)	0.383	17.3 (9.1–33.0)	<0.001

**Table 3:** Associated injuries of hospitalized trauma patients.

Variables	EMS RSI ≥ 1 ED RSI < 1 *n* = 85 (II)	EMS RSI < 1 ED RSI ≥ 1 *n* = 98 (III)	EMS RSI < 1 ED RSI < 1 *n* = 47 (IV)	EMS RSI ≥ 1 ED RSI ≥ 1 *n* = 3485 (I)	*OR (95%CI) p*		*OR (95%CI) p*		*OR (95%CI) p*	
					II *vs*. I		III *vs*. I		IV *vs*. I	
Head/Neck trauma										
Neurologic deficit	1 (1.2)	1 (1.0)	0 (0.0)	24 (0.7)	1.7 (0.2–12.8)	0.594	1.5 (0.2–11.1)	0.697	1.0 (1.0–1.0)	0.568
Cranial fracture	10 (11.8)	6 (6.1)	1 (2.1)	258 (7.4)	1.7 (0.9–3.3)	0.132	0.8 (0.4–1.9)	0.632	0.3 (0.0–2.0)	0.168
Epidural hematoma	5 (5.9)	4 (4.1)	2 (4.3)	173 (5.0)	1.2 (0.5–3.0)	0.701	0.8 (0.3–2.2)	0.691	0.9 (0.2–3.5)	0.824
Subdural hematoma	10 (11.8)	7 (7.1)	4 (8.5)	319 (9.2)	1.3 (0.7–2.6)	0.411	0.8 (0.4–1.7)	0.495	0.9 (0.3–2.6)	0.879
Subarachnoid hemorrhage	10 (11.8)	8 (8.2)	5 (10.6)	382 (11.0)	1.1 (0.6–2.1)	0.815	0.7 (0.4–1.5)	0.380	1.0 (0.4–2.5)	0.944
Intracerebral hematoma	6 (7.1)	1 (1.0)	0 (0.0)	69 (2.0)	3.8 (1.6–8.9)	0.001	0.5 (0.1–3.7)	0.498	-	0.330
Cerebral contusion	5 (5.9)	8 (8.2)	1 (2.1)	173 (5.0)	1.2 (0.5–3.0)	0.701	1.7 (0.8–3.6)	0.154	0.4 (0.1–3.0)	0.372
Cervical vertebral fracture	3 (3.5)	1 (1.0)	1 (2.1)	28 (0.8)	4.5 (1.4–15.2)	0.007	1.3 (0.2–9.5)	0.813	2.7 (0.4–20.2)	0.318
Maxillofacial trauma										
Orbital fracture	0 (0.0)	4 (4.1)	0 (0.0)	77 (2.2)	-	0.166	1.9 (0.7–5.3)	0.219	-	0.303
Maxillary fracture	0 (0.0)	1 (1.0)	1 (2.1)	54 (1.5)	-	0.248	0.7 (0.1–4.8)	0.674	1.4 (0.2–10.2)	0.750
Mandibular fracture	6 (7.1)	10 (10.2)	6 (12.8)	247 (7.1)	1.0 (0.4–2.3)	0.992	1.5 (0.8–2.9)	0.238	1.9 (0.8–4.6)	0.134
Nasal fracture	1 (1.2)	3 (3.1)	2 (4.3)	80 (2.3)	0.5 (0.1–3.7)	0.494	1.3 (0.4–4.3)	0.619	1.9 (0.5–7.9)	0.375
Thoracic trauma										
Rib fracture	11 (12.9)	17 (17.3)	14 (29.8)	357 (10.2)	1.3 (0.7–2.5)	0.419	1.8 (1.1–3.1)	0.023	3.7 (2.0–7.0)	<0.001
Hemothorax	3 (3.5)	6 (6.1)	4 (8.5)	48 (1.4)	2.6 (0.8–8.6)	0.099	4.7 (2.0–11.2)	<0.001	6.7 (2.3–19.3)	<0.001
Pneumothorax	4 (4.7)	2 (2.0)	3 (6.4)	57 (1.6)	3.0 (1.1–8.4)	0.031	1.3 (0.3–5.2)	0.756	4.1 (1.2–13.6)	0.012
Hemopneumothorax	5 (5.9)	2 (2.0)	6 (12.8)	35 (1.0)	6.2 (2.4–16.1)	<0.001	2.1 (0.5–8.7)	0.317	14.4 (5.8–36.2)	<0.001
Lung contusion	4 (4.7)	0 (0.0)	0 (0.0)	43 (1.2)	4.0 (1.4–11.3)	0.006	-	0.269	-	0.444
Abdominal trauma										
Intra–abdominal injury	4 (4.7)	1 (1.0)	7 (14.9)	77 (2.2)	2.2 (0.8–6.1)	0.127	0.5 (0.1–3.3)	0.426	7.7 (3.4–17.8)	<0.001
Hepatic injury	12 (14.1)	5 (5.1)	7 (14.9)	70 (2.0)	8.0 (4.2–15.4)	<0.001	2.6 (1.0–6.7)	0.035	8.5 (3.7–19.7)	<0.001
Splenic injury	4 (4.7)	2 (2.0)	4 (8.5)	28 (0.8)	6.1 (2.1–17.8)	<0.001	2.6 (0.6–11.0)	0.185	11.5 (3.9–34.2)	<0.001
Retroperitoneal injury	2 (2.4)	0 (0.0)	0 (0.0)	5 (0.1)	16.8 (3.2–87.7)	<0.001	-	0.707	-	0.795
Renal injury	2 (2.4)	0 (0.0)	1 (2.1)	13 (0.4)	6.4 (1.4–29.0)	0.005	-	0.545	5.8 (0.7–45.3)	0.057
Extremity trauma										
Humeral fracture	1 (1.2)	5 (5.1)	3 (6.4)	174 (5.0)	0.2 (0.0–1.6)	0.107	1.0 (0.4–2.6)	0.961	1.3 (0.4–4.2)	0.664
Radial fracture	5 (5.9)	7 (7.1)	3 (6.4)	351 (10.1)	0.6 (0.2–1.4)	0.203	0.7 (0.3–1.5)	0.340	0.6 (0.2–2.0)	0.403
Ulnar fracture	3 (3.5)	5 (5.1)	4 (8.5)	173 (5.0)	0.7 (0.2–2.2)	0.546	1.0 (0.4–2.6)	0.951	1.8 (0.6–5.0)	0.268
Pelvic fracture	6 (7.1)	4 (4.1)	7 (14.9)	121 (3.5)	2.1 (0.9–4.9)	0.078	1.2 (0.4–3.3)	0.746	4.9 (2.1–11.1)	<0.001
Femoral fracture	14 (16.5)	14 (14.3)	8 (17.0)	393 (11.3)	1.6 (0.9–2.8)	0.137	1.3 (0.7–2.3)	0.355	1.6 (0.8–3.5)	0.218
Tibial fracture	10 (11.8)	14 (14.3)	5 (10.6)	373 (10.7)	1.1 (0.6–2.2)	0.755	1.4 (0.8–2.5)	0.260	1.0 (0.4–2.5)	0.989
Fibular fracture	5 (5.9)	10 (10.2)	3 (6.4)	222 (6.4)	0.9 (0.4–2.3)	0.856	1.7 (0.9–3.3)	0.128	1.0 (0.3–3.3)	0.997

**Table 4:** Length of stay in the hospital and the intensive care unit.

Variables	EMS RSI ≥ 1 ED RSI < 1 *n* = 85 (II)	EMS RSI < 1 ED RSI ≥ 1 *n* = 98 (III)	EMS RSI < 1 ED RSI < 1 *n* = 47 (IV)	EMS RSI ≥ 1 ED RSI ≥ 1 *n* = 3485 (I)	*OR (95%CI) p*		*OR (95%CI) p*		*OR (95%CI) p*	
					II *vs*. I		III *vs*. I		IV *vs*. I	
Hospital LOS										
days	15.2 ± 14.5	10.9 ± 12.0	16.9 ± 16.2	9.5 ± 9.4	-	0.001	-	0.156	-	<0.001
ICU LOS										
n (%)	39 (45.9)	19 (19.4)	24 (51.1)	575 (16.5)	4.3 (2.8–6.6)	<0.001	1.2 (0.7–2.0)	0.448	5.3 (3.0–9.4)	<0.001
days	13.1 ± 14.3	6.5 ± 6.2	13.0 ± 20.3	7.6 ± 8.2	-	0.296	-	0.397	-	0.070

## Abbreviations

The following abbreviations are used in this manuscript:

AOR	Adjusted odd ratio
AIS	Abbreviated injury scale
CI	Confidence interval
ED	Emergency department
EMS	Emergency medical services
GCS	Glasgow coma scale
HR	Heart rate
ICU	Intensive care unit
ISS	Injury severity score
LOS	Length of stay
NISS	New injury severity score
OR	Odds ratio
RSI	Reverse shock index
SI	Shock index
SBP	Systolic blood pressure
TRISS	Trauma and injury severity score
